# Frontotemporal Lobar Degeneration‐TDP Type C With Striatal Glial Cytoplasmic Inclusions and Motor Neuron Degeneration

**DOI:** 10.1111/nan.70090

**Published:** 2026-07-10

**Authors:** Akiko Uchino, Kazutomi Kanemaru, Airi Tarutani, Masato Hasegawa, Hiroya Naruse, Hiroyuki Ishiura, Shigeo Murayama, Yuko Saito

**Affiliations:** ^1^ Department of Neuropathology (Brain Bank for Aging Research), Tokyo Metropolitan Geriatric Hospital and Institute of Gerontology Tokyo Japan; ^2^ Department of Preventive Medical Center Kitasato University Kitasato Institute Hospital Tokyo Japan; ^3^ Department of Neurology Tokyo Metropolitan Geriatric Hospital and Institute of Gerontology Tokyo Japan; ^4^ Department of Clinical Medical Sciences Tokyo Metropolitan Institute of Medical Science Tokyo Japan; ^5^ Department of Neurology, Graduate School of Medicine The University of Tokyo Tokyo Japan; ^6^ Department of Neurology, Graduate School of Medicine, Dentistry and Pharmaceutical Sciences Okayama University Okayama Japan

**Keywords:** amyotrophic lateral sclerosis, annexin A11, corticobasal syndrome, frontotemporal lobar degeneration, glial cytoplasmic inclusions, motor neuron disease, TDP‐43

## Abstract

- We report an autopsy case of FTLD‐TDP type C with severe striatal involvement with annexin A11 and pTDP‐43‐positive glial cytoplasmic inclusions.
- This case also showed both upper and lower motor neuron involvement.
- The patient developed progressive asymmetric rigidity accompanied by marked striatal atrophy.
- This case expands the recognised clinicopathological spectrum of FTLD‐TDP type C and may support the concept of an annexin A11–associated pathogenic continuum linking FTLD and ALS.

## Graphical abstract

We report an autopsy case of frontotemporal lobar degeneration (FTLD)‐TDP type C with severe striatal involvement and annexin A11‐ and phosphorylated TDP‐43‐positive glial cytoplasmic inclusions. The patient developed progressive asymmetric rigidity accompanied by marked striatal atrophy and showed both upper and lower motor neuron involvement. These findings expand the clinicopathological spectrum of FTLD‐TDP type C and may support the concept of an annexin A11‐associated pathogenic continuum linking FTLD and amyotrophic lateral sclerosis.

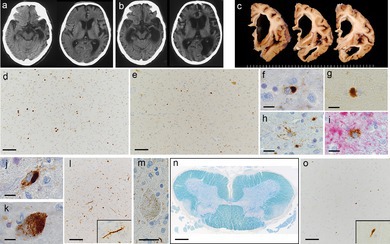

Frontotemporal lobar degeneration (FTLD) with TAR DNA‐binding protein of 43 kDa (TDP‐43) type C is pathologically defined by long dystrophic neurites (DNs) in the neocortex, typically with minimal neuronal cytoplasmic inclusions (NCIs) [1]. It is most commonly associated with semantic dementia (SD), and motor system involvement is considered to be rare [1]. Recently, annexin A11 has been found to co‐localise with phosphorylated TDP43 (pTDP‐43) inclusions in FTLD‐TDP type C [2, 3]. This observation has expanded our understanding of the histopathological characterisation of FTLD‐TDP type C and suggests that annexin A11 may play an important role in its pathogenesis. Annexin A11 aggregation is well recognised in amyotrophic lateral sclerosis (ALS) cases harbouring pathogenic variants in annexin A11 [4, 5]. Moreover, recent research has identified variants in annexin A11 as a cause of corticobasal syndrome (CBS), although neuropathological characterisation has not been reported [6]. The precise role of annexin A11 in FTLD, ALS and CBS remains to be elucidated; however, these disorders may share overlapping pathological mechanisms. Annexin A11 may therefore represent a potential mechanistic link across these clinicopathological entities. Here, we report a neuropathologically confirmed case of FTLD‐TDP type C with severe degeneration and numerous glial cytoplasmic inclusions (GCIs) in the striatum, as well as degeneration of upper and lower motor neurons. The present case initially presented with progressive semantic impairment and subsequently developed asymmetric rigidity. These findings expand the known histopathological spectrum of FTLD‐TDP type C and raise new questions regarding the role of glial pathology and annexin A11 in disease progression.

A 67‐year‐old man presented with difficulty in naming words and recollecting meanings of words. There was no family history of neurological disorders. Within a year, he developed fluent but meaningless speech, palilalia and impaired comprehension. There was no apparent muscle weakness, rigidity, tremor, or ataxia. A cerebrospinal fluid examination showed an elevated protein level of 85.5 mg/dL (cutoff value 45 mg/dL) with no increase in cell count. The electroencephalogram showed alpha waves without sharp waves or left–right sided predominance. Brain computed tomography (CT) findings showed left‐predominant temporal lobe atrophy (Figure 1a), and he was clinically diagnosed with SD. Two and a half years later, his speech was only in a meaningless voice. In addition, rigidity emerged in the right upper limb and gradually progressed to the lower limbs. Six years after onset, brain CT showed progressive temporal lobe atrophy and pronounced atrophy of the left caudate nucleus (Figure 1b). He died of pneumonia at 79 years, 12 years after symptom onset.

**FIGURE 1:**
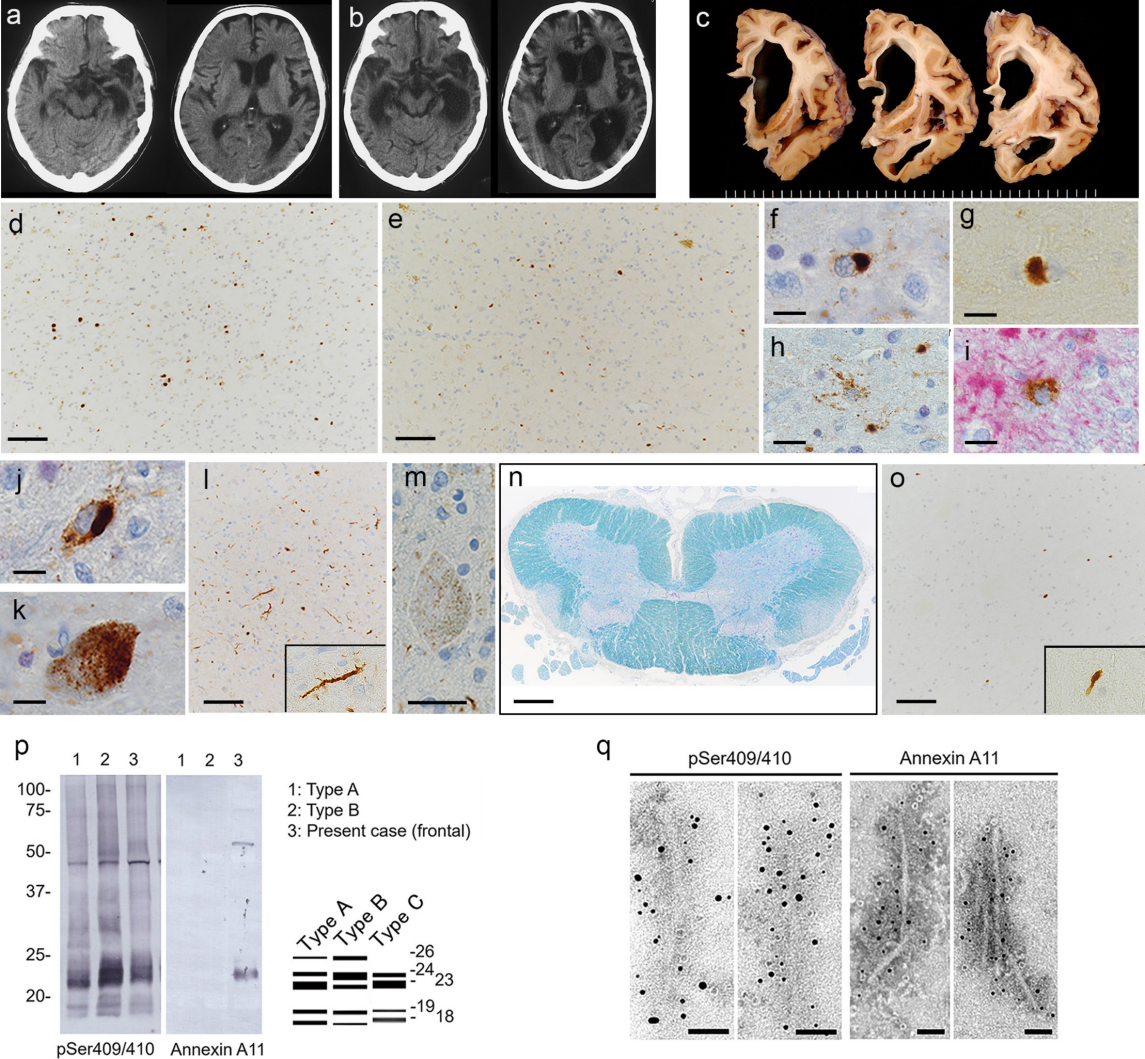
Radiological and neuropathological findings in the present case. (a) Brain computed tomography (CT) shows left‐predominant temporal lobe atrophy 1 year after onset. (b) Six years after onset, repeated brain CT shows progressive left‐dominant temporal lobe atrophy and left caudate nucleus atrophy. (c) Macroscopic findings reveal marked atrophy of the left temporal and frontal lobes, caudate nucleus and amygdala. (d,e) Numerous round‐shaped glial cytoplasmic inclusions (GCIs) immunoreactive for phosphorylated TDP‐43 (pTDP‐43) and annexin A11 in the putamen (d, pTDP‐43; e, annexin A11). (f–i) GCIs and neuronal cytoplasmic inclusions (NCIs) in the putamen. Round‐shaped GCIs immunoreactive for pTDP‐43 (f) and annexin A11 (g). Granular GCIs immunoreactive for pTDP‐43 (h). Double immunohistochemistry against pTDP‐43 (brown) and GFAP (red) shows co‐localisation for both protein in glia cell in the putamen (i). pTDP‐43 immunoreactive NCIs in a small neuron (j) and a large neuron (k). (l) In the primary motor cortex, there are many annexin A11‐immunoreactive dystrophic neurites (DNs). (m) pTDP‐43 immunoreactive granules in Betz cell. (n) Semi‐macroscopically, myelin pallor is present in the lateral corticospinal tracts in the cervical spinal cord. (o) In the anterior horn of lumbar spinal cord, sparse DNs and dot‐like structures immunoreactive for pTDP‐43 are found. (p) Western blot analysis shows a band of approximately 45 kDa corresponding to full‐length pTDP‐43 and C‐terminal fragments of 18, 19, 23 and 24 kDa in the present case, consistent with a typical frontotemporal lobar degeneration (FTLD)‐TDP type C band pattern (left panel). The results using an anti‐annexin A11 antibody showed a 56‐kDa band that corresponded to full‐length annexin A11 and additional bands at 25 and 23 kDa in the present case, while no bands were detected in FTLD‐TDP types A and B (right panel). (q) Electron micrographs show fibrous structures positive for pTDP‐43 or annexin A11, after labeling with secondary antibody conjugated to 5 nm gold particles. Scale bars = 50 μm (d,e,j,m), 10 μm (f–k,m), 1 mm (n), 50 nm (q).

Autopsy was performed 21 h after death, and neuropathological examination was performed as previously described [7]. The brain weighed 1157 g before fixation. Macroscopically, there was marked atrophy of the left temporal and frontal lobes, caudate nucleus and amygdala (Figure 1c). Microscopic examination revealed severe neuronal loss and gliosis in the temporal tip, transentorhinal cortex, as well as in the caudate nucleus and putamen. Immunohistochemistry for pTDP‐43 and annexin A11 revealed numerous DNs in the cortex and, more notably, numerous GCIs and NCIs in the striatum (Figure 1d,e). Most GCIs were rounded inclusions associated with small nuclei, morphologically reminiscent of those observed in amyotrophic lateral sclerosis (ALS) (Figure 1f,g). These inclusions were negative for Olig2 and MAP 2 (Supplementary Figure S1). A small subset of GCIs exhibited granular morphology with multiple processes (Figure 1h), some of which showed co‐localisation with GFAP (Figure 1i). NCIs were detected in both large‐ and small‐sized neurons (Figure 1j,k). The morphology of pTDP‐43 and annexin A11‐positive structures was almost identical, and the abundance of these structures was comparable or slightly lower in annexin A11‐positive inclusions than in TDP‐43 pathology (Table 1). Degeneration was also observed in the upper and lower motor system. In the primary motor cortex, Betz cell loss and gliosis were accompanied by many pTDP‐43‐ and annexin A11‐positive DNs (Figure 1l). Only a small number of Betz cells showed pTDP‐43‐positive granules (Figure 1m). In the spinal cord, myelin pallor was present in the corticospinal tracts (Figure 1n), while anterior horn cells were preserved, with mild gliosis. Bunina bodies were absent. In the spinal cord, aggregates of pTDP‐43 and annexin A11 were short DNs and dot‐like structures (Figure 1o).

**TABLE 1:** Semi‐quantitative evaluation of neuronal loss, gliosis, pTDP‐43 and annexin A11 positive structures.

	Neuronal loss	Gliosis	pTDP‐43	Annexin A11
Frontal cortex	+++	+++	+++	++
Temporal cortex (temporal tip)	+++	+++	++	++
Parietal cortex	+	+	++	+++
Occipital cortex	−	−	+	+
Primary motor cortex	+++	++	+++	++
Caudate nucleus	+++	+++	+++	++
Putamen	+++	+++	+++	++
Globus pallidus	+++	+++	+	+
Thalamus	−	−	+	+
Amygdala	+++	+++	+	++
Hippocampus	++	+++	+++	++
Entorhinal cortex	+++	+++	+++	++
Substantia nigra	−	−	−	−
Facial nucleus	−	−	−	−
Locus celureus	−	−	−	−
Hypoglossal nucleus	−	−	−	−
Inferior olivarly nucleus	−	−	−	−
Anterior horn	−	+	+	+
Pyramidal tract	na	++	−	−

*Note:* +++, abundant; ++, moderate; +, sparse; −, none.

Abbreviations: na, region not available; pTDP‐43, phosphorylated TAR DNA‐binding protein 43.

Western blot analysis of sarkosyl‐insoluble fractions from the frontal cortex showed full‐length pTDP‐43 (approximately 45 kDa) and its characteristic C‐terminal fragments (18, 19, 23 and 24 kDa), consistent with FTLD‐TDP type C (Figure 1p, left panel) [1]. Annexin A11 analysis revealed a 56‐kDa full‐length band and additional 23–25 kDa fragments, which were not observed in FTLD‐TDP type A or B (Figure 1p, right panel) [2, 3]. No pathogenic variants or expansions were identified in known ALS/FTLD‐related genes, including *ANXA11* and *C9orf72* [8]. Immunoelectron microscopy showed 10–15‐nm filaments double‐labelled with pTDP‐43 and annexin A11 antibodies, confirming heteromeric filament formation (Figure 1q) [2, 3].

This case highlights several key points. First, GCIs are rarely described in FTLD‐TDP type C, where the hallmark is DNs rather than glial pathology [1]. In the present case, most GCIs were negative for both Olig2 and MAP 2. MAP 2 negativity makes a neuronal origin less likely, although it cannot be completely excluded. Likewise, the absence of Olig2 immunoreactivity does not exclude an oligodendroglial origin, as Olig2 expression may vary according to the differentiation stage and pathological state of oligodendroglial lineage cells. A subset of GCIs showed co‐localisation with GFAP, indicating that at least some inclusions were present in astrocytic cells. To the best of our knowledge, no previous studies have specifically focused on GCIs in FTLD‐TDP type C. Notably, a recent study expanding the spectrum of annexin A11 proteinopathy reported that annexin A11–positive GCIs were not clearly observed in FTLD‐TDP and motor neuron disease [9]. In contrast, the abundance of annexin A11‐ and pTDP‐43‐positive GCIs in the striatum in the present case suggests that glial pathology may be under‐recognised and potentially more prevalent than currently appreciated. Such glial inclusions may represent either a distinct pathological variant or a feature associated with disease progression in certain cases. Furthermore, the involvement of anatomically connected cortical and subcortical regions raises the possibility that pTDP‐43 and annexin A11 pathology may propagate along axonal pathways, as has been proposed in ALS [10]. Nevertheless, it remains possible that the striatal glial pathology observed here is unique to the present case. Therefore, further studies focusing on glial pathology in FTLD‐TDP type C will be necessary to clarify its pathological and clinical significance.

Second, severe involvement of the striatum observed in the present case is considered to underlie the clinical presentation of progressive asymmetric rigidity. The involvement of the striatum in FTLD‐TDP type C has been documented in a limited number of cases [11]; however, the correlation between striatal involvement and clinical symptoms remains to be elucidated. Progressive asymmetric rigidity with behavioural change and speech impairment may be reminiscent of CBS, although the present case did not fulfill diagnostic criteria for CBS [12]. It is known that a subset of FTLD‐TDP cases can present with CBS [12], and pathogenic variants in annexin A11 have also been implicated in CBS [6]. These observations raise the possibility of a link between CBS and annexin A11‐related pathology.

Finally, the presence of motor neuron pathology in FTLD‐TDP type C further supports the concept of clinical and pathological overlap with ALS. TDP‐43 and/or annexin A11 pathology in motor neurons has been reported in a small number of FTLD‐TDP type C cases [13, 14, 15]. In most of these cases, the pathology predominantly involved upper motor neurons with only limited lower motor neuron involvement, which is similar to the findings in the present case. However, the morphology of TDP‐43/annexin A11 pathology appears to differ from that observed in ALS cases with annexin A11 variants. In ALS, lower motor neurons typically exhibit skein‐like NCIs, resembling those seen in sporadic ALS [4, 5]. These differences may suggest divergent pathogenic mechanisms despite the shared involvement of annexin A11 and TDP‐43. More recently, Ghayal et al. proposed three neuropathological phenotypes across the spectrum of FTLD and motor neuron disease [9]. Neuropathologically, the present case most closely resembles their phenotype 3, which is characterised by long neurites in the cortex and NCIs in the putamen and dentate gyrus. In that study, six cases were classified into this phenotype, and one of them clinically presented with CBS. Therefore, further detailed clinicopathological studies will be required to clarify the clinical and pathological correlations within this phenotype.

In conclusion, we described a case of FTLD‐TDP type C with glial pathology in the striatum and involvement of the motor system. The strong co‐localisation of annexin A11 and phosphorylated TDP‐43 across diverse lesion types and brain regions supports a mechanistic link between these proteins and suggests that annexin A11 may actively participate in TDP‐43 aggregation or propagation. The present case provides new insights into the morphology and distribution of TDP‐43/annexin A11 pathology and expands the recognised pathological spectrum of FTLD‐TDP type C, supporting the concept of an annexin A11–associated pathogenic continuum linking FTLD, ALS and CBS.

## Author Contributions

The first draft of the manuscript was written by Akiko Uchino and revised by Shigeo Murayama and Yuko Saito. Neuropathological analysis was performed by Akiko Uchino, Shigeo Murayama and Yuko Saito. Kazutomi Kanemaru collected clinical data. Airi Tarutani and Masato Hasegawa performed western blot and immunoelectron microscopic analysis. The genetic analysis was performed by Hiroya Naruse and Hiroyuki Ishiura. All authors read and approved the final manuscript.

## Funding

This work was supported by Japan Agency for Medical Research and Development (AMED) under Grant Numbers JP21wm0425019, JP25wm0625126 and JP24dk0207074h0001 (M.H.); by MEXT/JSPS KAKENHI under Grant Number JP22H04923 (CoBiA); and by the MHLW Research on Rare and Intractable Diseases Program under Grant Number JPMH23FC1008.

## Ethics Statement

All procedures performed in studies involving human participants were in accordance with the ethical standards of the institutional and/or national research committee and with the 1964 Helsinki Declaration and its later amendments or comparable ethical standards. This study was approved by the Ethics Committee of (No.2005‐16). Informed consent was obtained from the participant's family included in the study.

## Conflicts of Interest

The authors declare no conflicts of interest.

## Supporting information


**Figure S1:** Left panel: Double immunohistochemistry for pTDP‐43 (brown) and Olig2 (red), showing no co‐localisation of Olig2 with cells containing small rounded pTDP‐43‐positive inclusions. Right panel: Double immunohistochemistry for pTDP‐43 (brown) and MAP 2 (red), showing no co‐localisation of MAP 2 with cells containing small rounded pTDP‐43‐positive inclusions. Scale bar = 20 μm in the left panel, 50 μm in the left panel.

## Data Availability

The datasets used and/or analysed during the current study are available from the corresponding author upon reasonable request.
