# Supplementary figures and images for: Frontotemporal Lobar Degeneration‐TDP Type C With Striatal Glial Cytoplasmic Inclusions and Motor Neuron Degeneration
Source: Neuropathol Appl Neurobiol. 2026 Jul 10;52(4):e70090. doi: 10.1111/nan.70090 (PMC13352230; doi:10.1111/nan.70090)

**Supplementary Fig. 1**


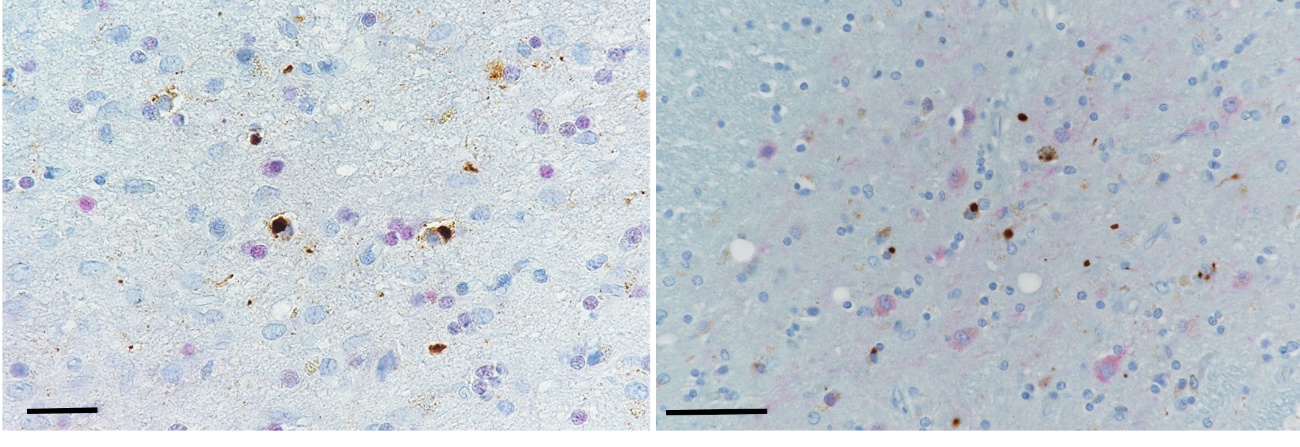

Supplement: Supplementary file 1 — Figure S1: Left panel: Double immunohistochemistry for pTDP‐43 (brown) and Olig2 (red), showing no co‐localisation of Olig2 with cells containing small rounded pTDP‐43‐positive inclusions. Right panel: Double immunohistochemistry for pTDP‐43 (brown) and MAP 2 (red), showing no co‐localisation of MAP 2 with cells containing small rounded pTDP‐43‐positive inclusions. Scale bar = 20 μm in the left panel, 50 μm in the left panel. [file NAN-52-e70090-s001.docx]
